# Does Watching a Play about the Teenage Brain Affect Attitudes toward Young Offenders?

**DOI:** 10.3389/fpsyg.2017.00964

**Published:** 2017-06-09

**Authors:** Robert Blakey

**Affiliations:** Centre for Criminology, University of OxfordOxford, United Kingdom

**Keywords:** neuroscience communication, neurocriminology, youth justice policy, public attitudes, moral responsibility, blame attribution

## Abstract

Neuroscience is increasingly used to infer the cognitive capacities of offenders from the activity and volume of different brain regions, with the resultant findings receiving great interest in the public eye. This field experiment tested the effects of public engagement in neuroscience on attitudes toward offenders. Brainstorm is a play about teenage brain development. Either before or after watching this play, 728 participants responded to four questions about the age of criminal responsibility, and the moral responsibility and dangerousness of a hypothetical young or adult offender. After watching the play, participants perceived the young offender as less likely to reoffend than the adult offender and the young, but not adult, offender as less morally responsible for his actions, especially on the first offense. Therefore, public engagement in the newest arrival to the criminological scene – neuroscience – may shift support for different youth justice responses.

## Introduction

According to one dominant interpretation of neurocriminology, offenders do not choose to commit crime (Greene and Cohen, 2004). Instead, people offend as a result of unusual characteristics of their brains. While this argument may appear controversial, it is merely the claim that no part of the conscious mind is entirely independent of genetic and environmental influences. Put simply, scientific explanations of behavior do not provide scope for a soul in causation (Kolber, 2016). Therefore, while ‘there are many causes that impinge on behavior’ (Greene and Cohen, 2004, p. 1781), ‘all of them…must exert their influence through the brain’ and hence, ‘all behavior is caused by our brains’ (Raine and Glenn, 2014, p. 161).

Importantly, the uncontrollability of the brain poses no implications for the plausibility of behavior change: the probability of caused offending can still be reduced as a result of natural changes to genetic development and deliberate changes to the environmental inputs to the brain (Dar-Nimrod and Heine, 2011). This study aimed to test the receptivity of lay people to this message in the context of young people; specifically, the message that adolescents are influenced by uncontrollable brain mechanisms that naturally develop, and so change, with age.

If people are not truly responsible for their brains, offenders may not be truly responsible for their crimes. In this respect, neurocriminology may threaten a critical foundation of the retributive justification for punishment: that offenders should be punished in proportion to their culpability and the seriousness of the crime committed (von Hirsch, 1976). The question of concern to the current study is *not* whether this reasoning is valid but whether lay people reason about neuroscience in this manner. Lay people are defined as ordinary members of the public who have no expertise in crime causation, neuroscience or legal attributions of culpability. Such individuals do not ordinarily consider the brain to be an important cause of offending (Furnham and Henderson, 1983; Gabbidon and Boisvert, 2012; Gajos et al., 2014).

### Does Exposure to Neuroscience Reduce Support for Punishment?

Instead of measuring punitiveness, the current study considered the mechanisms by which neuroscience could influence punitiveness; that is by changing the perceived moral responsibility and dangerousness of the described offender (Carlsmith and Darley, 2008). In research that has previously considered this question, it is possible to distinguish three approaches. The first approach – the approach of psychology – most closely represents the type of neuroscience presented in the current study: participants read a challenge to the existence of free will, before judging a specific offender. The second approach is that of experimental philosophy: participants are asked to *assume* that a challenge to free will is true, before judging a specific offender. The third approach is that of mock court studies: participants are asked to read a challenge to free will that directly relates to the specific offender, before judging that offender. Each approach has generated different findings; hence this paper begins by reviewing the conclusions of the three approaches, before relating those conclusions to the type of neuroscience presented in the current study.

### The Psychological Approach

Under the approach of psychology, Shariff et al. (2014) report four studies supporting the effect of challenging the existence of free will on the sentences recommended by lay people. The most striking effect was observed in study 2 (p. 3): after reading a short neurobiological explanation of behavior that explicitly rejected free will, the average participant recommended a sentence of half the length of a control group; this effect size was observed even though the described offender ‘beat a man to death’ – an emotive offense that would typically invite blame that was immune to claims of causation (Nahmias et al., 2007; Nichols and Knobe, 2007). The desired sentence length was also reduced after participants read that conscious decisions could be predicted from prior unconscious brain activity (Soon et al., 2008; Bode et al., 2011). In study 4 – the study that most closely represents the current study –, participants recommended a shorter sentence after (compared to before) completing one university term of introductory cognitive neuroscience; in contrast, no change in punitiveness was observed across a term of geography (study 4). Therefore, challenges to free will may reduce the desire for people to punish offenders on retributive grounds.

However, such findings failed to replicate in a similar and more recent series of studies (Monroe et al., 2017). Monroe et al. (2017) observed no effects of challenging belief in free will on attributions of wrongfulness, blame or punishment to the specific offender presented. These null effects persisted no matter how free will was challenged, which varied from asserting genetic and environmental causation, a supercomputer or brain scanner with infallible predictive powers, or the existence of determinism or a destiny. Likewise, the null effects persisted for offense types varying from sunbathing nude to selling drugs.

These null effects also cannot be attributed to the failure of the manipulation: belief in free will for the self and for people in general was successfully challenged, even though the attribution of free will to the specific offender was not. Hence challenges to generic belief in free will failed to inform attributions of blame and punishment, which, instead, correlated with perceptions of the specific offender as free, intentional and capable of choice. Hence these findings directly challenge the retributive relevance of distal causation in the public eye.

### The Philosophical Approach

The inconsistencies observed in the psychological evidence appear less surprising when those findings are placed in the broader context of experimental philosophy. Experimental philosophers also present participants with challenges to free will, yet, in addition, ask participants to assume the challenge accurately describes a hypothetical world or the real world (Nichols, 2011). In contrast to the psychological approach, therefore, experimental philosophers measure the perceived implications of causation rather than also the believability of causation. Even when participants are required to accept that no ultimate form of free will exists, the evidence tends to support the conclusion of Monroe et al. (2017): people continue to hold actors morally responsible for their actions in entirely caused universes, at least in judgements of specific immoral acts in the real world – the context of relevance to the current study (Nahmias, 2006; Nahmias et al., 2006, 2007; Nichols and Knobe, 2007; Roskies and Nichols, 2008).

The philosophical work suggests that public opinion is consistent with the normative legal response to neuroscience: the neurobiological roots of intent bear no implications for the legal attribution of responsibility (Morse, 2004, 2006, 2008; but see Kolber, 2016). The findings of experimental philosophy stake a similarly skeptical prediction: it may be plausible to convince people that the brain causes behavior, yet far less plausible to convince people that such a fact poses any implications for moral responsibility (Nahmias et al., 2014). As a strong and evolved instinct, retributive punitiveness may be relatively immune to reason (Fehr and Gächter, 2002; Crockett et al., 2014; Nadelhoffer et al., 2014).

### The Mock Court Approach

There remains one context in which people do appear to accept the implications of neuroscience for blame and punishment; that is when neuroscience is presented in direct relation to a *mentally ill* offender. There are two ways in which lay people could interpret evidence of mental disorder implicated in a criminal act: either as suggesting that the offender is unchangeably dangerous or that the offender is underserving of blame, thereby aggravating or mitigating the resultant sentence (Barth, 2007; Aspinwall et al., 2012). The historical abuse of biological criminology would predict the aggravating edge of this double-edged sword to be sharper (Rafter, 2008); indeed, genetic attributions do amplify the perceived dangerousness of psychotics (Angermeyer et al., 2011). However, a small body of experimental evidence suggests that the mitigating edge is sharper.

Lay people who are asked to act as judges in mock court scenarios attribute less moral responsibility to an offender whose mental illness is described in neurobiological, rather than cognitive, terms – both when the evidence is presented by the defense (Gurley and Marcus, 2008; Schweitzer and Saks, 2011; Schweitzer et al., 2011), by the defense but together with a prosecution that labels the defendant dangerous (Greene and Cahill, 2012), and even by the prosecution (Aspinwall et al., 2012). The net mitigating effect of neuroscience has also been observed with real judges engaged in mock sentencing (Aspinwall et al., 2012) and an analysis of 800 real cases in which neuroscience has been presented (Denno, 2015). Such findings lend support to Greene and Cohen (2004): people may recognize neurobiological dispositions to offend as undermining the culpability of offenders, unlike cognitive and social dispositions to offend (Dar-Nimrod et al., 2011).

This mock court research falls against a backdrop of studies into the ‘seductive allure’ of neuroscience. Beyond the criminal justice context, people find psychological explanations more compelling when those explanations are supplemented with irrelevant neuroscience (Weisberg et al., 2008, 2015). Note this is an effect of neuroscientific description, not of brain images *per se* (Gruber and Dickerson, 2012; Hook and Farah, 2013; Schweitzer et al., 2013; Fernandez-Duque et al., 2015; but see McCabe and Castel, 2008; Farah and Hook, 2013), as observed in studies of 1,971 participants (Michael et al., 2013). Importantly, the appeal of neuroscience cannot be attributed to the addition of jargon or the status of neuroscience (Fernandez-Duque et al., 2015), yet may represent a broader effect of reducing mechanisms down to their smaller parts (Hopkins et al., 2016). Therefore, the influence of neuroscientific defenses in court may represent the broader influence of neuroscientific arguments.

Similar to mock court researchers, experimental philosophers also directly relate causation to the specific offender, yet still, fail to effectively challenge attributions of blame. Hence one might speculate that the ‘success’ of the mock court approach arises from the fact that, in these cases, neuroscience is used to cast doubt on the free will of the *mentally ill* offender. This generates two implications: first, neuroscience may successfully cast doubt on the capacity of the offender to make a conscious, intentional and desired choice (hereon termed a rational choice), rather than to exercise an uncaused free will. Indeed, lay attributions of moral responsibility are highly sensitive to conscious intent (e.g., Alicke, 1992; Shepherd, 2012) and desire (Woolfolk et al., 2006; Monroe et al., 2017); people even tend to define free will as the capacity for rational choice (Monroe and Malle, 2010).

Second, people may be more willing to grant mitigating influence to neuroscience that appears restricted in its relevance. In the mock court scenarios, the neuroscience concerns only one particular offender with a rare form of severe mental illness; in this context, the applicability of the science is very narrow. Indeed, people are more likely to relinquish blame given evidence of causation when the evidence is applied to a hypothetical world (Nahmias et al., 2007; Roskies and Nichols, 2008) or when people judge complete causation to be inapplicable to the real world (Shepherd, 2012). Put simply, people may be more willing to accept implications of neuroscience that are restricted in scope. The rare psychotic offender and every rational actor on Earth represent two extremes of applicability; hence this study aimed to consider a middle ground: can neuroscience influence judgements of adolescents, whose capacity to exercise rational choice is diminished, yet in a normal developmental manner?

### The Role of Neuroscience in Youth Justice

The current study tested the effects of lay people learning about teenage brain development on the attribution of responsibility and dangerousness to young offenders. In the United States, expert testimony regarding adolescent brain development has informed youth justice cases in the Supreme Court, including Roper v. Simmons (2005), Graham v. Florida (2010) and Miller v. Alabama (2012) (Feld, 2013; Steinberg, 2013). Although the current study tested the effects of communicating neuroscience in a theater (rather than a courtroom), such seminal cases provide close parallels to the intervention and measures used in the present study.

The neuroscience presented in the current study conveyed the consistent message of a large body of relevant evidence (Blakemore and Choudhury, 2006), summarized by Laurence Steinberg in the following manner: ‘the teenage brain is like a car with a great accelerator but terrible brakes. With powerful impulses under poor control, the likely result is a crash’ (Buchen, 2012, p. 305). In more scientific terms, the brain region implicated in reward processing – the nucleus accumbens – responds more to rewards in adolescents (than in adults), yet the brain region implication in self-control – the orbitofrontal cortex – is less responsive (Galvan et al., 2006). Hence adolescents are more in need of a degree of self-control that they don’t have (Harden and Tucker-Drob, 2011). Therefore, in part, youth offending may reflect the reduced capacity, rather than the reduced willingness, of young offenders to exercise rational choices (Steinberg and Scott, 2003; Steinberg, 2009, 2012).

Neuroscience may bolster the argument that impulsivity reflects an impairment in capacity, rather than a lack of motivation, and thereby reduce the punitiveness of lay people. Indeed, punitiveness toward young offenders is predicted by the perceived comparability of adolescents and adults in respect to moral responsibility (Allen et al., 2012), intentionality and naivety (Metcalfe et al., 2015). Since lay people are therefore sensitive to concerns of relevance to adolescence neuroscience, the current study sought to test their response to this science.

### The Plastic Brain

There is one message about the adolescent brain that researchers have yet to introduce to the public, given their focus on the brain as a deterministic force; that is the message that the brain is changeable (or plastic). By default, people conceive of brain-based traits as the best indicators of moral character (Fernandez-Duque and Schwartz, 2016). Hence Snead (2007, p. 274) predicts that the net effect of neuroscience will be aggravating: by suggesting that offenders have unchangeably dangerous characters, ‘the criminal regime desired by cognitive neuroscientists would, tragically and ironically, prove far harsher and less humane’ – a prediction that is grounded in the historical abuse of neuroscience to justify eugenics (Rafter, 2008). While Appelbaum and Scurich (2014) observed the aggravating effects of genetic descriptions, the aggravating effect of neuroscientific description is absent from the limited evidence collected so far (Aspinwall et al., 2012; Saks et al., 2014).

The current study questions whether the aggravating interpretation of neuroscience is inevitable. Miles (2013) suggests that although neuroscience is communicated without reference to fate, lay people may infer fatalism from ‘merely’ deterministic messages; lay people may infer that offenders are predestined to offend from evidence that their prior criminal behavior was caused, especially if caused by biology (Dar-Nimrod and Heine, 2011). From this perspective, people construe challenges to free will as challenges to self-control, thereby eliminating the potential for a change in environments or beliefs to pull the offender from a predestined path.

If lay people do infer fatalism from neuroscience, this would suggest lay people consider the brain to be entirely uncontrollable. Indeed, people do not consider the brain sufficient or necessary to attribute agency to artificial actors, such as God, robots, frogs, newborns, or organizations (Gray et al., 2007; Knobe and Prinz, 2008). People also withhold attributions of choice from neurobiological actors who appear unconscious or unintentional (Nahmias et al., 2007). Such evidence suggests that in judgements of artificial actors, people do not represent the brain as a factor that we always control or the only factor over which control can be exercised. However, a different finding emerges when participants are asked to judge real human beings: people judge more brain-based traits to be more controllable (Fernandez-Duque and Schwartz, 2016). Hence, in this indirect respect, people appear to represent the brain as controllable.

One might reconcile the conflicting findings by speculating that people are willing to consider the brain uncontrollable when there is no incentive to consider the brain controllable – and there is no such incentive in judgements of artificial actors. In contrast, there are moral incentives for people to consider the moral characteristics of real brains controllable (Fernandez-Duque and Schwartz, 2016). The controllability of moral traits provides a compelling justification for blaming immoral actors and crediting moral actors. Extending this claim, Pitts-Taylor (2010, p. 640) forecasts a neuroscientific society in which mental ‘health maintenance becomes a responsibility or a duty rather than a right’ (Pitts-Taylor, 2010, p. 639). Accordingly, offenders could be held responsible for ‘failing’ to maintain their neurobiological health. Hence people might seek to deem the brain controllable to this alarming neoliberal extent.

Collectively, this research suggests that people have incentives to consider brain-based moral traits both controllable *and* stable, since this perception would provide grounds for punishment on the basis of blame *and* dangerousness. This raises the question of how receptive people would be to the message that the brain is neither controllable nor stable; that is the message conveyed by the neuroscience presented in the current study. Specifically, participants learnt that the adolescent brain lacks the capacity to exercise rational control over strong impulses, yet also develops that capacity naturally with age. Hence, in the current study (unlike prior research), the communicated neuroscience explicitly challenged the tendency for people to infer fate from biological explanation (Dar-Nimrod and Heine, 2011; Miles, 2013). Consequently, this was the first study to present the brain as changeable (or plastic). Therefore, this study tested the scope for people to perceive the adolescent brain as uncontrollable yet unstable, and subsequently, to perceive the young offender as less blameworthy yet more capable of reform.

### The Current Study

This study measured the effects of exposure to a neurobiological explanation of adolescent behavior on attitudes toward a hypothetical young or adult offender who committed a serious assault three times in 3 years. Participants judged the offender both after the first offense and after the third offense, and made those judgements either before or after watching Brainstorm – a play about teenage brain development. The study extended prior research by three means: first, by presenting neuroscience as relevant to a group of people – teenagers – rather than everyone or only psychotic offenders, second, by presenting the brain as unstable, and third, by presenting neuroscience in a far more engaging format than the passages of text typically used in previous research.

### Hypotheses

Three hypotheses were proposed on the basis of two assumptions:

A1: Participants would be more likely to attribute impulsive adolescent offending to uncontrollable yet unstable brain mechanisms after watching the play.
A2: Since the play was about the adolescent brain, the play would only change attitudes toward young, not adult, offenders.

In combination, the predicted outcomes of these two assumptions generated three hypotheses:

H1: The play would increase the age of criminal responsibility that was perceived to be most appropriate.
H2: The play would reduce attributions of moral responsibility to a hypothetical young, but not adult, offender.
H3: The play would reduce the perceived probability of a young, relative to adult, offender reoffending.

## Materials and Methods

### Design

This field experiment adopted a mixed model design, with the persistence of the offending manipulated within groups, and exposure to neuroscience and the age of the described offender manipulated between groups to avoid order effects; this was necessary since it was impossible to counter-balance exposure to neuroscience.

### Participants

The 728 participants responded to four questions about crime either before or after watching Brainstorm at the National Theater in London. The study was conducted around all six performances of the play in 2016; those were one matinee and five evening performances from Tuesday 29th March to Saturday 2nd April 2016. Given that 1,320 people watched the play across this period, the survey response rate was 55%. Since it was necessary for the survey to be very short, it was impossible to collect demographic information on the participants.

### The Play

Brainstorm is a play about teenage brain development, directed by Ned Glasier and Emily Lim. The play was produced by Company Three (formally known as Islington Community Theater) in collaboration with two cognitive neuroscientists. It was presented at the Park Theater in January 2015 and subsequently at the National Theater in July 2015 and March 2016. The play was also adapted for BBC iPlayer. Further information about the play, including a link to purchase a copy of the script, is available at www.companythree.co.uk/brainstorm. The script conveys two primary messages that the audience could relate to young offending:

(1) The teenage brain is not a dysfunctional version of the adult brain. The teenage brain has evolved to generate behaviors that facilitate development, such as risk-taking and self-consciousness.
(2) In adolescence, the limbic system – the brain region that renders risk-taking rewarding – develops far more quickly than the prefrontal cortex – the brain region that enables people to form intentions, anticipate consequences and exercise self-control. Hence teenagers can behave like a car without brakes, exhibiting behavioral displays of emotion that adults would ordinarily inhibit, such as aggression.

### Procedure

During the 90 min before the play began, people were handed one of two envelope types upon arrival at the theater foyer. The envelopes differed in the text printed on the front: the text either instructed participants to open the envelope before watching the play or after watching the play; only the latter type of envelope was sealed. Each envelope contained a pencil and a survey that either described a hypothetical young or adult offender. Although participants were instructed to respond privately, it was anticipated that people might talk to each other about the survey; hence everyone who arrived at the theater in the same group received the same survey type to minimize the likelihood of participants learning of the differences between survey types.

One of the four survey types was randomly allocated to participants by six research assistants. Each assistant held a box containing four bags of envelopes – one bag for each survey type – and systematically rotated between each bag in the process of distributing envelopes. One might fear that the process of random allocation was undermined by differences in the response rate between conditions. For example, people may have required greater interest in adolescence neuroscience or young offenders to be sufficiently motivated to complete the survey after the play. By condition, the response rates were 61% (the young offender before the play; *N* = 200), 44% (the adult offender before the play; *N* = 146), 59% (the young offender after the play; *N* = 195) and 57% (the adult offender after the play; *N* = 187). Hence the response rate was reduced only for judgements of the adult offender before the play.

### Survey Items

First, participants were asked: ‘What do you think should be the age of criminal responsibility – the minimum age at which people can be arrested and charged with a crime?’ (Q1). The subsequent three questions were oriented around a short vignette about a hypothetical offender called Adam: ‘Adam commits a serious assault (a physical attack) against a stranger who has insulted him. Adam is [14 or 44] years old and has never committed a crime before.’ Participants were asked ‘To what extent do you think Adam is morally responsible for committing this crime?’ (1 = not at all responsible, 9 = entirely responsible; Q2). Subsequently, the vignette continued: ‘Adam commits another serious assault in similar circumstances at the age of [15 or 45] and then another at the age of [16 or 46]. From 1 to 9, to what extent do you think Adam is morally responsible for committing this crime on the third occasion?’ (Q3). Finally, participants were instructed to ‘imagine that Adam is never caught or punished and nobody tries to stop his criminal behavior. What do you think is the probability that Adam will commit another serious assault in his [30s or 60s]?’ (Q4).

## Results

I excluded values that fell more than three times the interquartile range from the mean (Q1: N = 3) and missing or illegible responses (Q1: N = 10, Q2: N = 3, Q3: N = 4, Q4: N = 11).

### The Recommended Age of Criminal Responsibility

Participants recommended a significantly higher age of criminal responsibility after the play (*M* = 15.89, *SD* = 2.61) compared to before the play (*M* = 15.14, *SD* = 2.51), *t*(713) = 3.92, *p* < 0.001, *d* = 0.29 (hypothesis 1). The lowest of these mean recommendations – the mean before watching the play – differed significantly from the current age of criminal responsibility in England and Wales (10), as indicated by a one-sample *t*-test, *t*(339) = 37.73, *p* < 0.001, *d* = 4.10.

### Attributions of Moral Responsibility

Attributions of moral responsibility were analyzed using a mixed-model ANOVA, with the Age of the offender [Young, Adult] and the Play [Before, After] as between-subjects factors, and the Number of the offense [First, Third] as a within-subjects factor. Participants attributed significantly less moral responsibility to the young offender (*M* = 6.52, *SD* = 1.55) compared to the adult offender (*M* = 7.97, *SD* = 1.19), *F*(1,720) = 196.53, *p* < 0.001, $\eta_{p}^{2}$ = 0.21, to the offender upon his first offense (*M* = 6.57, *SD* = 1.93) compared to his third offense (*M* = 7.79, *SD* = 1.54), *F*(1,720) = 485.05, *p* < 0.001, and to the offender after the play (*M* = 7.09, *SD* = 1.64) compared to before the play (*M* = 7.28, *SD* = 1.50), *F*(1,720) = 5.39, *p* = 0.021, $\eta_{p}^{2}$ = 0.007.

**FIGURE 1:**
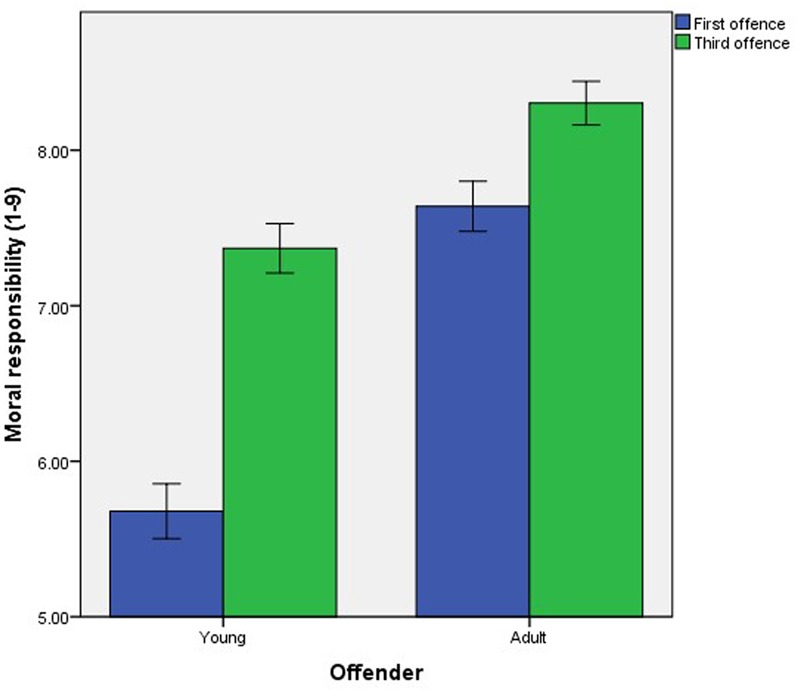
The effect of offense number on mean attributions of moral responsibility to the hypothetical young and adult offenders. Error bars represent the standard error.

There was no significant three-way interaction (*p* = 0.147). However, there were significant two-way interactions, which were explored using simple effects analyses. The first interaction was between Age and Number, *F*(1,720) = 93.48, *p* < 0.001, $\eta_{p}^{2}$ = 0.12. Participants attributed significantly more moral responsibility to the young and adult offender upon his third offense (young: *M* = 7.37, *SD* = 1.60, adult: *M* = 8.30, *SD* = 1.29) compared to his first offense (young: *M* = 5.68, *SD* = 1.78, adult: *M* = 7.64, *SD* = 1.49), young: *t*(393) = 23.87, *p* < 0.001, *d* = 1.00, adult: *t*(329) = 8.27, *p* < 0.001, *d* = 0.47. Although the increase in moral responsibility attributed for the third (compared to the first) offense was larger for the young (compared to the adult) offender (**Figure 1**), the increase remained significant for both the young and adult offender.

**FIGURE 2:**
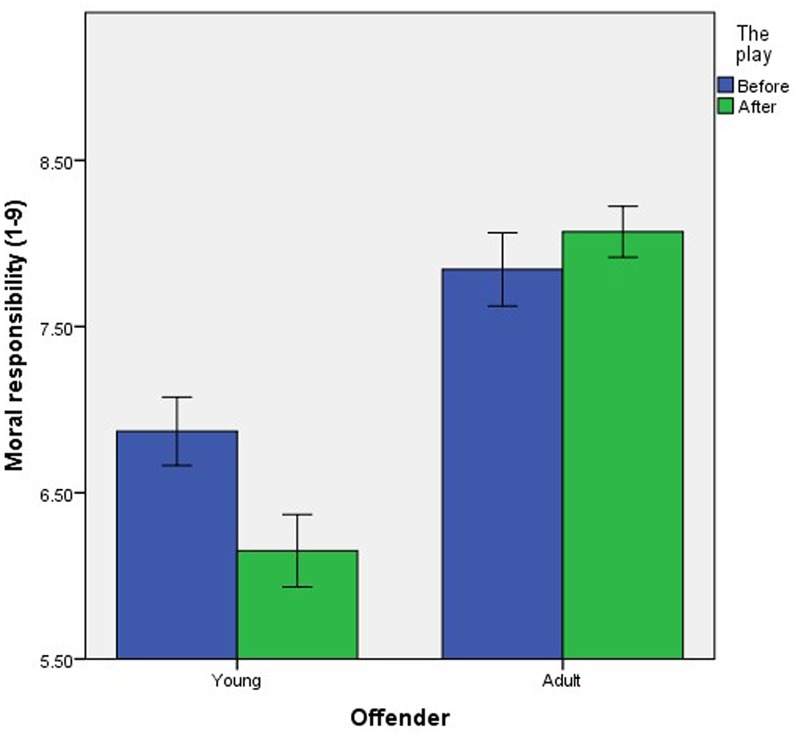
The effect of exposure to neuroscience on mean attributions of moral responsibility to the hypothetical young and adult offenders. Error bars represent the standard error.

The second interaction was between Age and Play, *F*(1,720) = 20.49, *p* < 0.001, $\eta_{p}^{2}$ = 0.03 (**Figure 2**). Participants attributed significantly less moral responsibility to the young offender after the play (*M* = 6.15, *SD* = 1.54) compared to before the play (*M* = 6.87, *SD* = 1.48), *t*(393) = 4.73, *p* < 0.001, *d* = 0.48 (hypothesis 2). In contrast, there was no significant difference between the attribution of moral responsibility to the adult offender before (*M* = 7.84, *SD* = 1.34) and after (*M* = 8.07, *SD* = 1.06) the play, *t*(266.76) = 1.67, *p* = 0.097 (equality of variance not assumed, *p* = 0.002).

**FIGURE 3:**
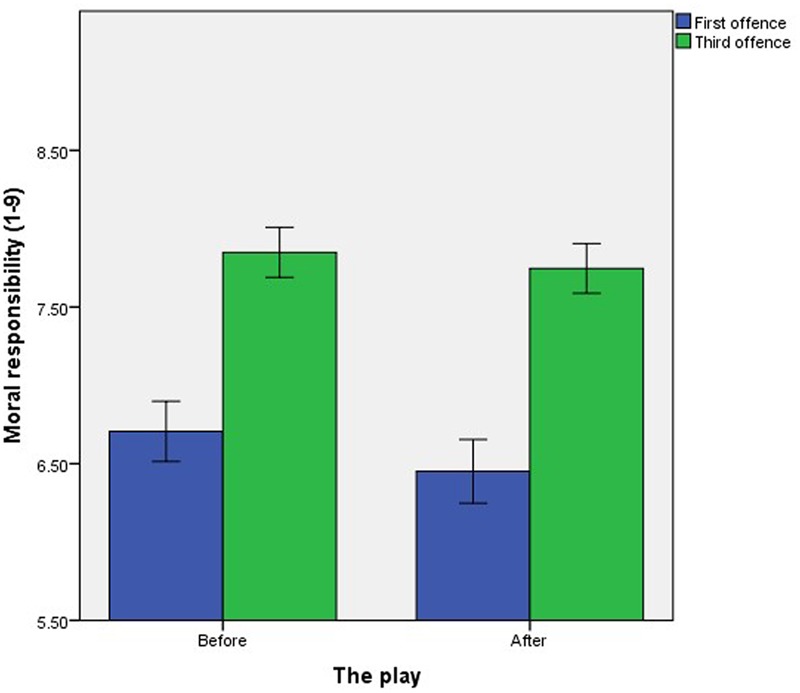
The effect of offense number on mean attributions of moral responsibility by exposure to neuroscience. Error bars represent the standard error.

The third interaction was between Number and Play, *F*(1,720) = 3.91, *p* = 0.048, $\eta_{p}^{2}$ = 0.005. Participants attributed significantly more moral responsibility to the offender upon his third offense compared to his first offense both before the play (first offense: *M* = 6.71, *SD* = 1.81, third offense: *M* = 7.85, *SD* = 1.51) and after the play (first offense: *M* = 6.45, *SD* = 2.01, third offense: *M* = 7.75, *SD* = 1.57), before: *t*(343) = 14.49, *p* < 0.001, *d* = 0.68, after: *t*(379) = 16.13, *p* < 0.001, *d* = 0.72. Although the increase in moral responsibility attributed for the third (compared to the first) offense was larger after the play (compared to before; **Figure 3**), the increase remained significant both before and after the play.

### Estimated Probability of Reoffending

Estimated probabilities of reoffending were analyzed using an independent-groups ANOVA, with Age [Young, Adult] and Play [Before, After] as between-subjects factors. Participants perceived the adult offender as more likely to reoffend in his 60s (*M* = 75.54, *SD* = 23.22) than the young offender in his 30s (*M* = 69.09, *SD* = 26.63), *F*(1,713) = 10.80, *p* = 0.001, $\eta_{p}^{2}$ = 0.02.

**FIGURE 4:**
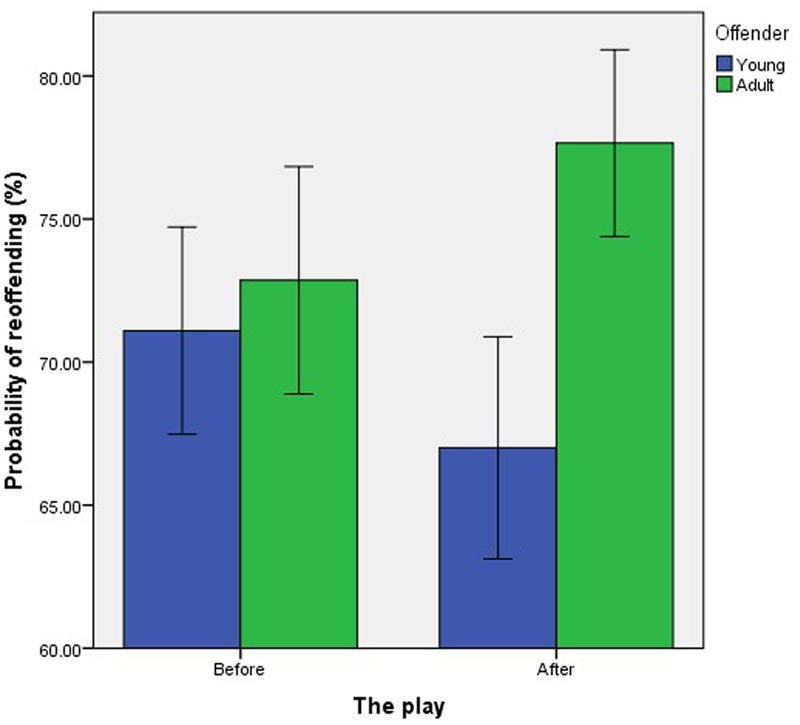
The effect of exposure to neuroscience on the perceived probability of the hypothetical young and adult offenders reoffending. Error bars represent the standard error.

There was also a significant interaction effect of Age and Play, *F*(1,713) = 5.54, *p* = 0.019, $\eta_{p}^{2}$ = 0.008. Before the play, there was no significant difference between the perceived probability of the young (*M* = 71.10, *SD* = 25.90) and adult (*M* = 72.86, *SD* = 24.12) offender reoffending, *t*(341) = 0.64, *p* = 0.523. However, after the play, participants perceived the young offender as significantly less likely to reoffend (*M* = 67.01, *SD* = 27.28) than the adult offender (*M* = 77.65, *SD* = 22.32), *t*(364.31) = 4.14, *p* < 0.001, *d* = 0.43 (equality of variance not assumed, *p* = 0.001; **Figure 4**; hypothesis 3).

## Discussion

After watching a play about teenage brain development, participants recommended a slightly higher age of criminal responsibility (hypothesis 1) and attributed less moral responsibility to a hypothetical young, but not adult, offender (hypothesis 2). These findings suggest that the play reduced the perceived culpability of young offenders – a core component of the retributive justification for punishment (von Hirsch, 1976).

Prior research has tended to present neuroscience either in relation to everyone (Nahmias et al., 2007) or in relation to a particular offender together with the diagnosis of a specific, rare and severe mental illness of salient relevance to the criminal behavior (Gurley and Marcus, 2008; Schweitzer and Saks, 2011; Schweitzer et al., 2011; Aspinwall et al., 2012; Greene and Cahill, 2012; Saks et al., 2014). This contrasts sharply with the means of presenting neuroscience in the current study: first, neuroscience was used to explain the behavior of an entire group of people – adolescents – and therefore invited the participants to accept the relevance of neuroscience to this entire group rather than only a rare type of defendant. Second, the relevance of neuroscience was less salient than in the mock court or philosophical research: the neuroscience was not presented in respect to the described offender; instead, the participants spontaneously inferred the relevance of the play to the vignette, given the age of the offender. Third, the brain was explicitly presented as changing across development, thereby countering attributions of stability to brain-based traits (Fernandez-Duque and Schwartz, 2016) and more generally, biology (Dar-Nimrod and Heine, 2011; Miles, 2013). The current study most resembles study 4 of Shariff et al. (2014) in these three respects.

Hence, together with Shariff et al. (2014), this study indicates the effects of public engagement in neuroscience beyond the context of mock court cases that fail to represent the average defendant. This study therefore supports the prediction of Greene and Cohen (2004) that neuroscience will undermine the attributions of responsibility that justify retributive punishment – yet only to a minimal extent. Though admittedly the intervention was only 70 min in duration, it exerted only small effects on attributions of responsibility.

The participants also did not generalize the presented neuroscience, failing to apply their understanding of the teenage brain to the brains of adult offenders. One might, however, question whether it would be reasonable to expect participants to equate the cause of teenage aggression depicted in the play to the cause of serious assault described in the vignette. The fact that the 44-year-old had refrained from offending for 44 years may have also undermined the perceived plausibility that the brain was responsible for the adult offending: if a seemingly stable factor, such as the brain, was to blame, one may have expected the 44-year-old to have offended earlier in his adult life. The outgroup ethnicity of the actors and actresses may have also undermined the receptivity of the audience to neuroscience as an explanation of ingroup behavior.

Independent of the play, participants attributed greater moral responsibility to the adult, compared to the young, offender (Scott et al., 2006), and for the third, compared to the first, offense. The large effect of offense number was observed for both the young and adult offenders, though to a greater extent for the young offender. However, this may reflect a ceiling effect: for the repeat adult offender, participants may have reached the maximum degree of responsibility that one would ever seek to attribute to a perpetrator of serious assault (see Alicke, 1992). Repeat offending can be interpreted in one of two ways: either as suggesting that the criminal behavior is more automatic or more deliberate; either as motivated by influences beyond the conscious control of the offender (von Hirsch, 2009) or as motivated by a stubborn intent to offend despite knowing the circumstances that generate the offending – and therefore the circumstances that could be avoided – and the harm inflicted as a result (Roberts, 2009). The distinction between these two interpretations is critical because the former corresponds to a mitigating and the latter an aggravating interpretation of persistent offending.

The aggravating interpretation prevailed in the current study, corroborating previous evidence that prior convictions increase lay attributions of guilt (Greene and Dodge, 1995), intentionality and culpability (Kliemann et al., 2008), and liability and dangerousness (Sanderson et al., 2000). The current participants attributed a recidivist premium to repeat offenders, especially when the offender was expected to have matured with age. Indeed, the mitigating influence of youth declines quickly as the age of the offender increases (Scott et al., 2006), for example, from 16 (Hough and Roberts, 2004) to 18 (Roberts and Hough, 2011). Therefore, in the current study, participants may have expected the hypothetical offender to have matured, and so to have gained moral responsibility, from the age of 14–16, but not from the age of 44–46.

It is important to note that the same participants judged the first and third offense and therefore, the manipulation of offense number was confounded by age (14–16 and 44–46). This combined manipulation accurately represents the fact that real judges consider the prior offenses of the same offender at an earlier age. Nevertheless, future research is advised to use an independent groups design to manipulate each factor separately and thereby test the effects of prior convictions independent of age.

Since participants were informed that the offending persisted despite the absence of punishment or any other intervention, the recidivist premium documented in this study appears to reflect the perceived intent of the offender. While an additional recidivist premium might be observed for any failure of the offender to respond to censure (Roberts, 2009), this cannot explain the current findings: the offender was described as having received no censure – no punishment or other intervention.

While there was a large increase in the attribution of moral responsibility for the third (compared to the first) offense both before and after the play, the increase was slightly greater after the play. According to attribution theory (Heider, 1958), one would expect people to attribute persistent offending to a stable feature of the offender that persisted with age (Carroll et al., 1987; Feather and Souter, 2002). While people might ordinarily perceive the brain-based traits as stable (Fernandez-Duque and Schwartz, 2016), akin to genes (Dar-Nimrod and Heine, 2011), the play explicitly presented the brain as developing across adolescence and therefore, as unstable. Hence participants may have sought a stable attribution for persistent offending, yet believed the brain failed to provide this attribution type: when the offender continued to offend despite the development of his brain, it may have become less believable that his brain caused the offending. People may also be less willing to excuse the repeat offender, instead seeking to attribute greater intent to account for the greater harm caused (e.g., Alicke, 1992; Roberts, 2009), thereby justifying greater punishment. Such predictions may be of interest to future research.

This study was the first to empirically test the relationship between attributions and the perceived probability of reoffending. Participants believed the young offender was less likely to reoffend than the adult offender only after watching the play (hypothesis 3). This is a striking result, given the theoretical proposal that neuroscience might actually amplify the perceived dangerousness of offenders (Snead, 2007); indeed, genetic attributions for psychosis do magnify the perceived dangerousness of psychotics (Angermeyer et al., 2011). The reversal of this effect in the current study may reflect the fact that this play depicted the brain as the site of behaviourally relevant change with age, thereby challenging the perception of biology as unchanging (Dar-Nimrod and Heine, 2011). Therefore, while this study did not document ‘public excitement about brain plasticity’ (Pitts-Taylor, 2010, p. 636), it did document receptivity to the idea.

By promoting attributions of criminal behavior to the brain and presenting the teenage brain as changing, the play may have motivated the reasoning that young people will desist from offending even without punishment or intervention. In fact, evidence suggests that diverting young offenders away from the CJS actually promotes desistance (McAra and McVie, 2007). The current participants believed the young offender was less likely to reoffend than the adult offender only after watching the play. For the public to support diversionary policies in youth justice, it may first be necessary to increase lay belief in the age-crime curve. Hence I predict that the play could bolster support for diversion relative to intervention or punishment – a hypothesis for testing by future research.

It is also interesting to note that before watching the play, the recommended age of criminal responsibility (15.26) was far higher than the actual age of criminal responsibility in England and Wales (10). This discrepancy may be attributed to lay belief in the value of diversion or the perception that even teenagers lack sufficient culpability to be arrested and charged. If lay people reason the latter, future research may wish to establish which source of culpability is deemed legally relevant to criminal responsibility (von Hirsch, 2001; Steinberg and Scott, 2003). According to empirical desert theory (Robinson and Darley, 1997), the legitimacy of the youth justice system may be challenged by this discrepancy between the desired and actual age of criminal responsibility, with potential implications for compliance. However, this finding may fail to generalize beyond the narrow subset of the population that attended this play – participants who likely sought to understand, rather than simply condemn, teenage behavior.

A clear limitation of this study is that there was no control condition; no condition in which participants watched a play about cognitive or social (rather than brain) development. Hence one could attribute the observed effects to the mere act of watching a play; that is engaging in a positive social experience. However, it is unclear how this account could explain the direction of the effects. Instead, therefore, a more substantive limitation regards the inability to specify which features of the play produced the observed effects; for example, it is possible that the same effects would have been observed after presenting any explanation of teenage behavior that induced greater empathy, perspective-taking, awareness or memory of adolescent experiences. Nevertheless, the plausibility of this account is challenged by the lack of strong evidence that even years of exposure to social explanations of crime erodes punitiveness (Selke, 1980; Giacopassi and Blankenship, 1991; Mackey and Courtright, 2000; Lambert, 2004; Shelley et al., 2011; Falco and Martin, 2012; Chen and Einat, 2015).

While this study necessarily lacked the control conditions and mediating measures that would be found in a mock court experiment, it gained ecological validity as a result of presenting neuroscience in a far more engaging format – a play that participants had chosen to attend. Hence this study extended previous research by considering a potentially more effective means of eroding punitive intuitions. Nevertheless, it is also possible that the attitudes of the current sample were more susceptible to neuroscientific change; that by purchasing tickets for the play, the sample had already expressed a desire to explain teenage behavior through neuroscience. Therefore, the current sample may have been unrepresentative on a theoretically important dimension.

In sum, exposure to neuroscience appeared to erode two bases of punitiveness: the perceived deservingness (Carlsmith and Darley, 2008) and the perceived dangerousness of the hypothetical offender (Maruna and King, 2009), especially the latter. While this suggests that neuroscience exerts a mitigating rather than aggravating influence (Aspinwall et al., 2012), generalization of the erosion process beyond young offenders and the first offense was limited. Such limits may reflect the strength of the punitive instinct (Fehr and Gächter, 2002; Nadelhoffer et al., 2013) and the resultant resistance of punitive intuitions to deliberative change (Garland, 2001).

More generally, this study suggests that lay people are willing to divorce their attributions of stability and controllability: participants were receptive to the message that adolescents are influenced by uncontrollable, yet also unstable, brain mechanisms. This characterisation of the brain represents the flip side of the default perception that brain-based traits are controllable yet also stable (Fernandez-Duque and Schwartz, 2016). Hence attributions of stability are not an unavoidable side-effect of attributions of uncontrollability; the consequentialist aggravating interpretation of neuroscience is not an unavoidable side-effect of the retributive mitigating interpretation. In communications that emphasize brain plasticity, such as this play, the public can make scientifically valid inferences from neuroscience.

## Ethics Statement

This study was carried out in accordance with the recommendations of the University of Oxford Central University Research Ethics Committee with implied consent gained from subjects. The use of implied consent was justified by the practical needs of the unique setting in which this study was conducted: a theater. Since the study involved answering only four questions, the use of implied consent allowed participants to take part without disrupting their experience as theater goers. The protocol was approved by the University of Oxford Central University Research Ethics Committee.

## Author Contributions

The study was designed, implemented and reported by RB.

## Conflict of Interest Statement

The author declares that the research was conducted in the absence of any commercial or financial relationships that could be construed as a potential conflict of interest.
